# Hypotensive and antihypertensive effects of an aqueous extract from Guinep fruit (*Melicoccus bijugatus* Jacq) in rats

**DOI:** 10.1038/s41598-020-75607-3

**Published:** 2020-10-29

**Authors:** Chukwuemeka R. Nwokocha, Alexia Gordon, Javier Palacios, Adrian Paredes, Fredi Cifuentes, Sheena Francis, JeAnn Watson, Rupika Delgoda, Magdalene Nwokocha, Ruby Alexander-Lindo, Rory Thompson, Donna Minott-Kates, Momoh A. Yakubu

**Affiliations:** 1 Department of Basic Medical Sciences, Faculty of Medical Sciences, The University of the West Indies, Mona Campus, Kingston 7, Jamaica; 2 Laboratorio de Bioquímica Aplicada, Departamento Química y Farmacia, Facultad de Ciencias de la Salud, Universidad Arturo Prat, 1110939 Iquique, Chile; 3 Departamento Química y Farmacia, Facultad de Ciencias Básicas, Universidad de Antofagasta, 1271155 Antofagasta, Chile; 4 Laboratorio de Fisiología Experimental, Instituto Antofagasta (IA), Universidad de Antofagasta, 1270300 Antofagasta, Chile; 5 Natural Products Institute, Faculty of Science and Technology, The University of the West Indies, Mona, Kingston 7, Jamaica; 6 Department of Pathology, Faculty of Medical Sciences, University of the West Indies, Mona Campus, Kingston 7, Jamaica; 7 Department of Chemistry, University of the West Indies, Mona, Kingston 7, Jamaica; 8 Department of Environmental and Interdisciplinary Sciences, College of Science, Engineering and Technology, Texas Southern University, Houston, TX 77004 USA

**Keywords:** Plant sciences, Hypertension

## Abstract

*Melicoccus bijugatus* Jacq (Mb) has been reported to have cardiovascular modulatory effects. In this study, we evaluated the antihypertensive effects and mechanism of action of Mb on N^G^-Nitro-l-arginine Methyl Ester (l-NAME) and Deoxycorticosterone Acetate (DOCA) rat models. Aqueous extract of Mb fruit (100 mg/kg) was administered for 6 weeks to rats by gavage and blood pressure was recorded. Effects of the extract on vascular reactivity was evaluated using isolated organ baths, and tissues were collected for biochemical and histological analysis. The systolic blood pressure (SBP), diastolic blood pressure (DBP) and mean arterial pressure (MAP) were significantly (*P* < 0.05) reduced with extract (100 mg/kg) administration and treatment compared to the hypertensive models. Mb (100 µg/mL) reduced the vascular contractility induced by phenylephrine (PE), and caused a dose-dependent relaxation of PE-induced contraction of aortic vascular rings. The vasorelaxation properties seemed to be endothelium dependent, as well as nitric oxide (NO) and guanylyl cyclase, but not prostaglandin dependent. Histomicrograph of transverse sections of the ventricles from the Mb group did not show abnormalities. The extract significantly (*P* < 0.05) reduced an l-NAME induced elevation of cardiac output and Creatine Kinase Muscle-Brain (CKMB), but had no significant impact on the activities of arylamine *N*-acetyltransferase. In conclusion, Mb significantly decreased blood pressure in hypertensive models. The extract possesses the ability to induce endothelium dependent vasodilation, which is dependent on guanylyl cyclase but not prostaglandins.

## Introduction

*Melicoccus bijugatus* Jacq is an edible jelly-like fruit belonging to the soapberry family, *Sapindaceae*. It is commonly referred to as Guinep, Spanish Lime, Quenepa, and it is native to the Americas and the Caribbean^1^. *M. bijugatus* is an excellent source of glucose, fructose, cellulose and vitamin A, which boosts the immune system and prevents the formation of urinary stones. As well as, vitamin C, which is a great antioxidant^2^, it is also famed to reduce the blood pressure^3,4^.

Hypertension is regarded as a severe cardiovascular risk factor with severe economic implications especially in developing countries. It becomes timely and important to pharmacologically validate and scientifically explore traditional remedies and folkloric use of natural plant products, to ascertain their efficacies, and validate their mechanisms of actions in the management of disease.

The fruit pulp and seeds contains a variety of phytochemicals compounds like epicatechin, catechin and procyanidin B_2_. Other active ingredients include phenols and naringenin (flavonoid) with antioxidant and anti-inflammatory properties. Phenolics such as caffeic and coumaric acid components may be the cause for its use in the management of asthma, diarrhea and hypertension, as they possess antiplatelet and antioxidant abilities. Caffeic acid is reported to inhibit vascular smooth muscle cell proliferation in rats induced by angiotensin II and selectively inhibits the biosynthesis of leukotriene, while saponins are reported to lower cholesterol^2^. Resveratrol, a constituent of the extract, is reported to inhibit nuclear factor kappa-light-chain-enhancer of activated B cells (NF-kB), a transcription factor involved in the inflammatory process^2^. Halberstein and Saunders^5^, and Facey et al.^6^ had reported that this fruit is used in the management of cardiovascular ailments in the Caribbean.

*Melicoccus bijugatus* possess cardio-protective properties, as it ameliorates isoproterenol induced myocardial injury. These cardiovascular effects may be due to the presence of phenolic acids, terpenes, fatty acids, and one glycosylated flavonoid constituents, reported with UHPLC high-resolution orbitrap mass spectrometry (UHPLC-OT-HR-MS) analysis. These are reported to confer a cardioprotective effect^7^. The effects of this plant extract on animal hypertensive models, and its possible mechanism of action is as yet to be ascertained.

The aim of the present investigation was to examine the effects of the aqueous extract of *M. bijugatus* in normotensive rats, it’s antihypertensive effect on DOCA-salt and l-NAME hypertensive animal models and possible mechanisms of action using in-vivo and in-vitro techniques. The impact of key enzymes like Creatine kinase muscle-brain (CKMB), High-sensitivity C-reactive protein (HS-CRP + CRP), Creatine kinase muscle-brain (CKMB), concentration of cardiac troponin I (cTnL), myoglobin (Myo), cardiac biomarkers associated with numerous cardiovascular disease states^8,9^, and arylamine *N*-acetyltransferase (NAT), a phase II drug metabolizing enzyme were evaluated to ascertain toxicity and possible cardio protection^10,11^.

## Results

### Effect of the administered extract on the systolic, diastolic, mean arterial pressure, pulse pressure and heart rate

**Figure 1:**
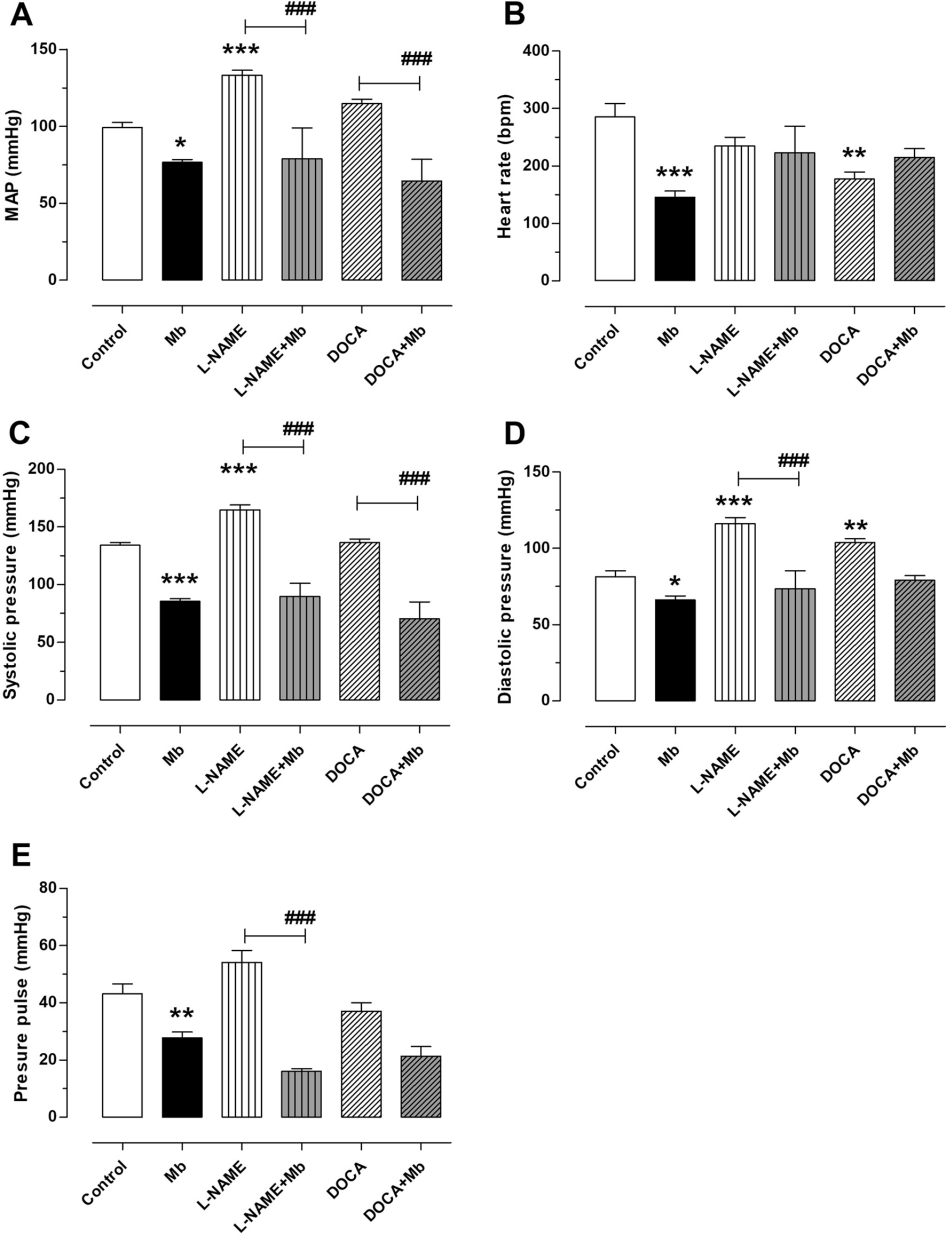
Effect of Mb (100 mg/kg) on MAP (**A**), HR (**B**), on Systolic Blood Pressure (SBP; **C**), Diastolic Blood Pressure (DBP; **D**) and Pulse Pressure (PP; **E**) of normotensive rats and hypertensive-induced with l-NAME or DOCA. Values are mean ± standard error of the mean (SEM). Statistically significant differences: *P < 0.05, ***P < 0.001 versus Control; ^###^*P < *0.001 versus l-NAME or DOCA. n = 4.

Results showed a significantly lower MAP in the Mb treated group compared to the control. As shown Fig. 1, *M. bijugatus* extract significantly (*P < *0.05) reduced MAP (76 ± 3 mmHg), SBP (85 ± 2 mmHg), and DBP (66 ± 3 mmHg) when compared to the control group, which had higher values of MAP (99 ± 3 mmHg), SBP (134 ± 2 mmHg), and DBP (81 ± 4 mmHg). The extract in this case reduced basal blood pressure in the control group, which had received no hypertensive inducing agent.

A significant *P < *0.001 decrease in HR was also observed in the Mb treated group compared with the control group (285 ± 24 bpm vs. 145 ± 11 bpm, respectively) (Fig. 1B). This observation is suggestive of the hypotensive properties of *M. bijugatus* and also its regulatory effects on MAP and HR.

*Melicoccus bijugatus* extract significantly (*P < *0.05) lowered the high blood pressures of DOCA-salt hypertensive models: MAP (65 ± 14 mmHg in DOCA + Mb vs 115 ± 3 mmHg in DOCA) a 43% decrease, SBP (70 ± 15 mmHg in DOCA + Mb vs 136 ± 3 mmHg in DOCA) a decrease of 48%, and DBP (79 ± 3 mmHg in DOCA + Mb vs 104 ± 2 mmHg in DOCA) a decrease of 24% (Fig. 1).

There were no significant changes in HR of controls compared with the DOCA-salt group (255 ± 40 bpm in DOCA vs 274 ± 32 bpm in DOCA + Mb) a slight decrease of 6% (Fig. 1B). The same can also be said when comparing Pulse Pressure (PP) in both groups. These results signify the effects of the extract in the l-NAME and DOCA-salt groups.

The l-NAME and DOCA-salt groups had DBP greater than the control group. The Mb treated l-NAME and DOCA-salt groups were found to have a lower DBP than the hypertensive untreated groups. In addition, the Mb extracts significantly (*P < *0.05) lowered the blood pressures of the l-NAME-induced hypertensive models (Fig. 1): MAP (79 ± 20 vs 133 ± 3 mmHg) a decrease of 40%, SBP (90 ± 20 mmHg l-NAME + Mb vs 165 ± 4 mmHg) a 45% decrease, DBP (73 ± 12 mmHg vs 116 ± 4 mmHg) a 31% decrease. Figure 1B showed that *M. bijugatus* significantly (*P < *0.001) lowered heart rate (HR) in the Mb group but had no effect on DOCA-salt group and l-NAME hypertensive model. However, a great difference was found in PP with l-NAME only having a PP of 54 ± 4 mmHg compared with the l-NAME + Mb treated group (16 ± 1 mmHg).

The Cardiac Output (CO) and Peripheral Resistance (PR) were calculated in accordance with the formulae: CO = Stroke Volume (PP) × HR, and PR = MAP/CO in relative units. It is known that stroke volume is proportional to pulse pressure^12,13^. As shown in Table 1, the CO decreased significantly in both MB treated hypertensive models, while, PR significantly decreased in normotensive rats.

**Table 1:** Effect of *M. bijugatus* (Mb; 100 mg/kg) on cardiac output (CO), and peripheral resistance (PR) in normotensive rats and hypertensive models with l-NAME or DOCA-salt.

	Normotensive rat		Hypertensive rat			
	Control	Mb	l-NAME	l-NAME + Mb	DOCA	DOCA + Mb
CO	0.15 ± 0.02	0.19 ± 0.01	0.23 ± 0.02	0.07 ± 0.0^###^	0.21 ± 0.02	0.12 ± 0.03^#^
PR	643 ± 21	402 ± 10***	577 ± 14	1100 ± 162^###^	547 ± 13	523 ± 114

***P < 0.001 vs. Control; ^#^*P* < 0.05, ^###^*P* < 0.001 vs. l-NAME or DOCA. CO and PR were measured in relative units. Values are mean ± SEM of 4 experiments.

### Electrocardiogram (ECG) and heart rate variability (HRV)

**Figure 2:**
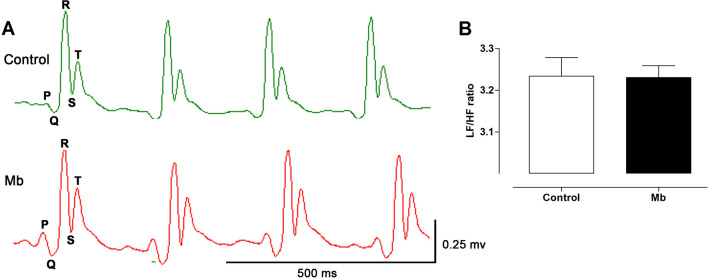
Effect of treatment with Mb (100 mg/kg) for 28 days on electrocardiogram (ECG) compared to control group (**A**), and sympathovagal balance of the ECG in normotensive rats (**B**). Values are mean ± SEM. n = 4.

Sympathovagal balance or the heart rate variability (HRV) of the ECG (Fig. 2A) was expressed as LH/HF ratio (LF, low frequency; HF, high frequency). *M. bijugatus* did not alter HRV (LF/HF) in normotensive rats (3.23 ± 0.04 control versus 3.23 ± 0.03 with 100 mg/kg Mb; Fig. 2B).

### Effect of M. bijugatus on relaxation of the aorta

**Figure 3:**
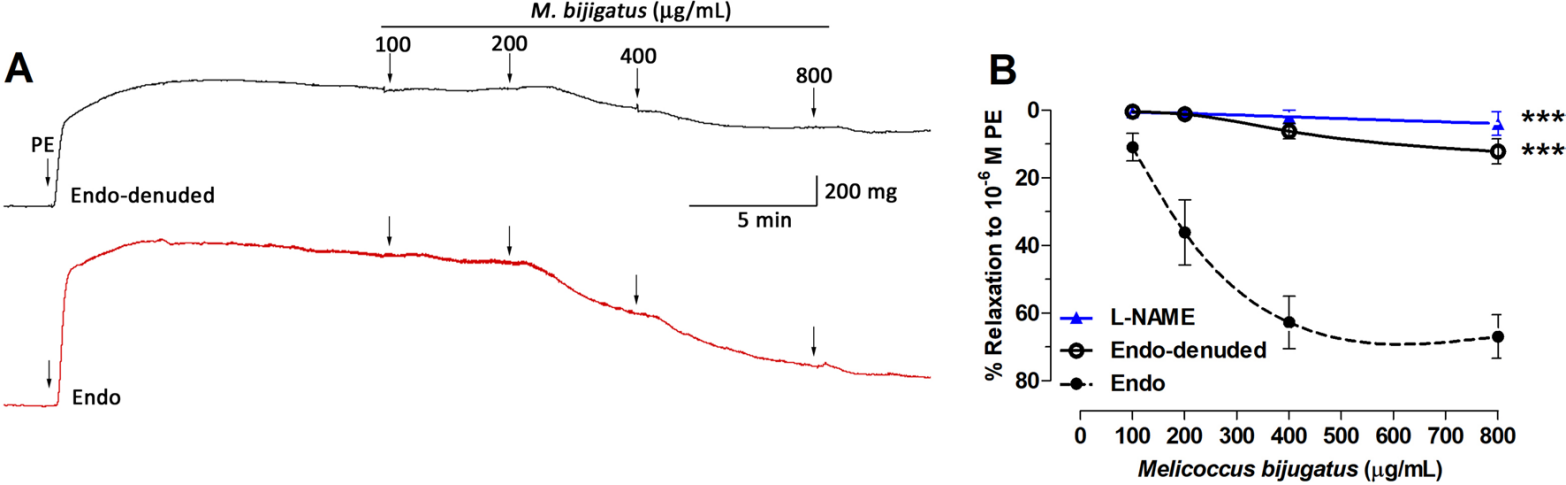
Concentration–response curves for the relaxation induced by the *M. bijugatus* extract on PE (10^–6^ M) pre-contracted rat aortic rings. Different protocols were used for intact of endothelium (Endo), absence of endothelium (Endo-denuded) (**A**), and in presence of l-NAME (10^–4^ M) (**B**). The responses are expressed as % of maximum PE-induced contraction. Each data point represents the mean ± SEM. ***P < 0.001 vs. Endo; n = 5.

**Figure 4:**
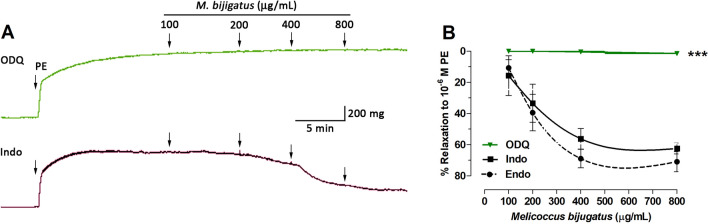
Effect of the guanylyl cyclase and prostaglandins on relaxation induced by the *M. bijugatus* extract on PE (10^–6^ M) pre-contracted rat aortic rings. The aortic rigs were pre-incubated with a specific soluble guanylyl cyclase inhibitor (ODQ; 10^–6^ M) and a non-selective cyclooxygenase inhibitor, indomethacin (Indo; 10^–5^ M) for 20 min before the experiment (**A**). The responses are expressed as % of maximum PE-induced contraction (**B**). Each data point represents the mean ± SEM. ***P < 0.001 vs. Endo n = 5.

The aqueous fruit extract of *M. bijugatus* caused a dose-dependent relaxation of intact aortic rings pre-contracted with phenylephrine (PE) with increasing doses (Fig. 3). The maximum relaxation to PE-induced contraction was 67 ± 6% in aorta with intact endothelium. In endothelium-denuded aortic rings, pre-incubated with l-NAME or 1H-(1,2,4)oxadiazolo[4,3-a]quinoxalin-1-one (ODQ; a specific soluble guanylyl cyclase inhibitor) the vasodilator effect of the Mb extract was completely abolished (Figs. 3, 4). However, the pre-incubation of aortic rings with indomethacin (a nonselective inhibitor of cyclooxygenases) did not affect the relaxation effect of the Mb extract (Fig. 4).

### Effect of M. bijugatus on PE-induced contraction

**Figure 5:**
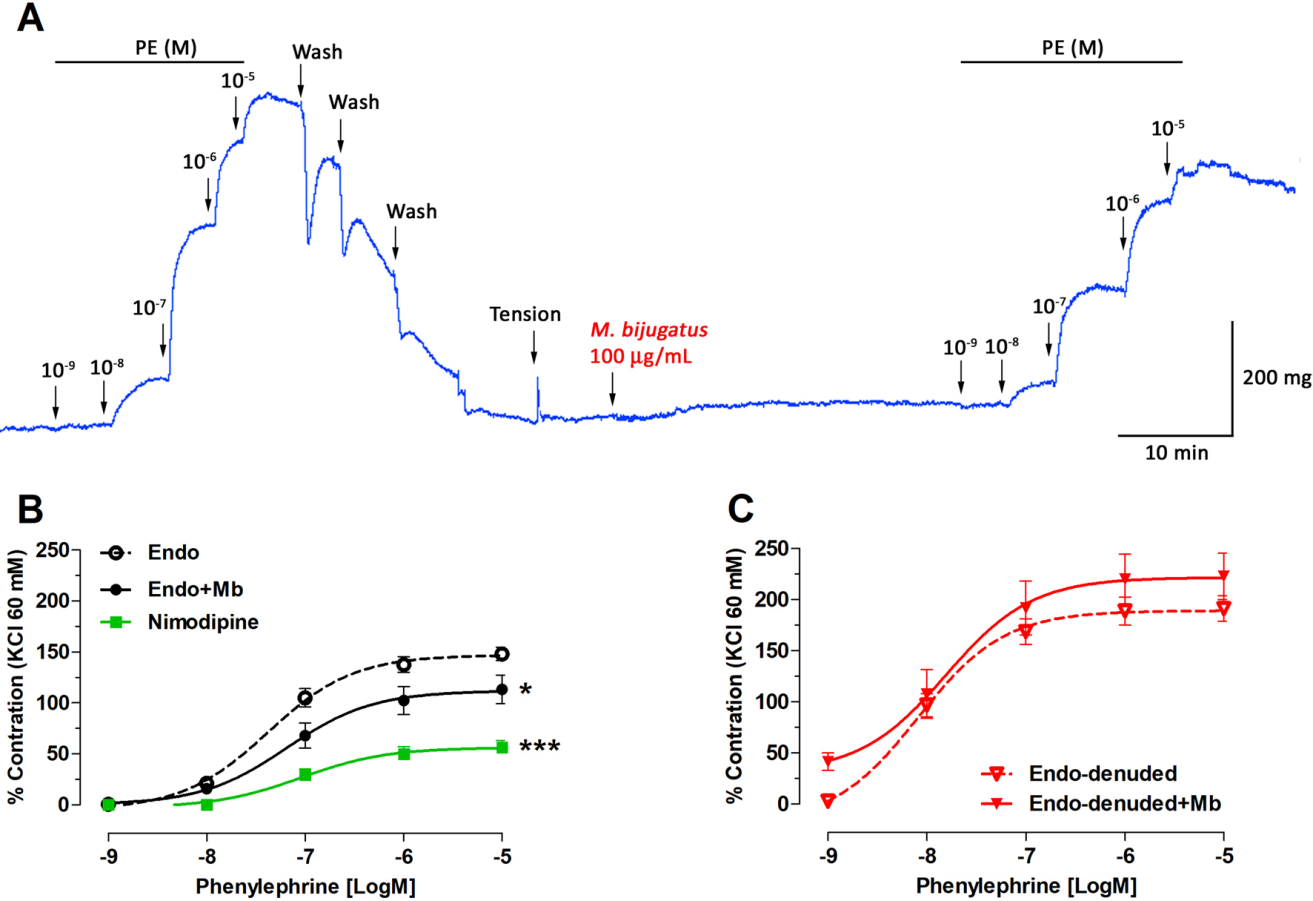
Original trace showing effect of *M. bijugatus* (Mb) on contractile response to PE (**A**). The responses are expressed as % of maximum PE-induced contraction in intact (Endo) (**B**) and denuded-rat aorta (Endo-denuded) (**C**). The tissues were pre-incubated with *M. bijugatus* (100 µg/mL) for 20 min before adding PE (10^–9^ to 10^–5^ M). Each data point represents the mean ± SEM. *P < 0.05, ***P < 0.001 vs. Endo; n = 5–7.

The aqueous extract of *M. bijugatus* did not have any vasoconstrictor effect when the aortic rings were incubated with 100 µg/mL Mb. However, the Mb caused a significant (*P* < 0.05) reduction in PE-induced contraction of intact aortic rings with a maximum contraction of 113 ± 14% (Endo + Mb) versus 148 ± 7% (Endo) and a rightward shift of the dose–response curve (Fig. 5A,B). The sensitivity (pD_2_) to PE in the presence of *M. bijugatus* (7.39 ± 0.18) was not significantly reduced when compared with the Endo (7.09 ± 0.17).

In addition, nimodipine (a blocker of L-type voltage-gated Ca^2+^ channels) significantly reduced the contractile response to PE (57 ± 7% Endo; *P* < 0.001) versus Endo curve.

On the other hand, the removal of endothelium in rat aorta significantly increased (*P* < 0.05) the contractile response to PE versus intact rat aorta (Fig. 5C). But, pre-incubation with Mb did not reduce the contractile response to PE, confirming that the presence of vascular endothelium is necessary for Mb effect.

### Histological analysis

**Figure 6:**
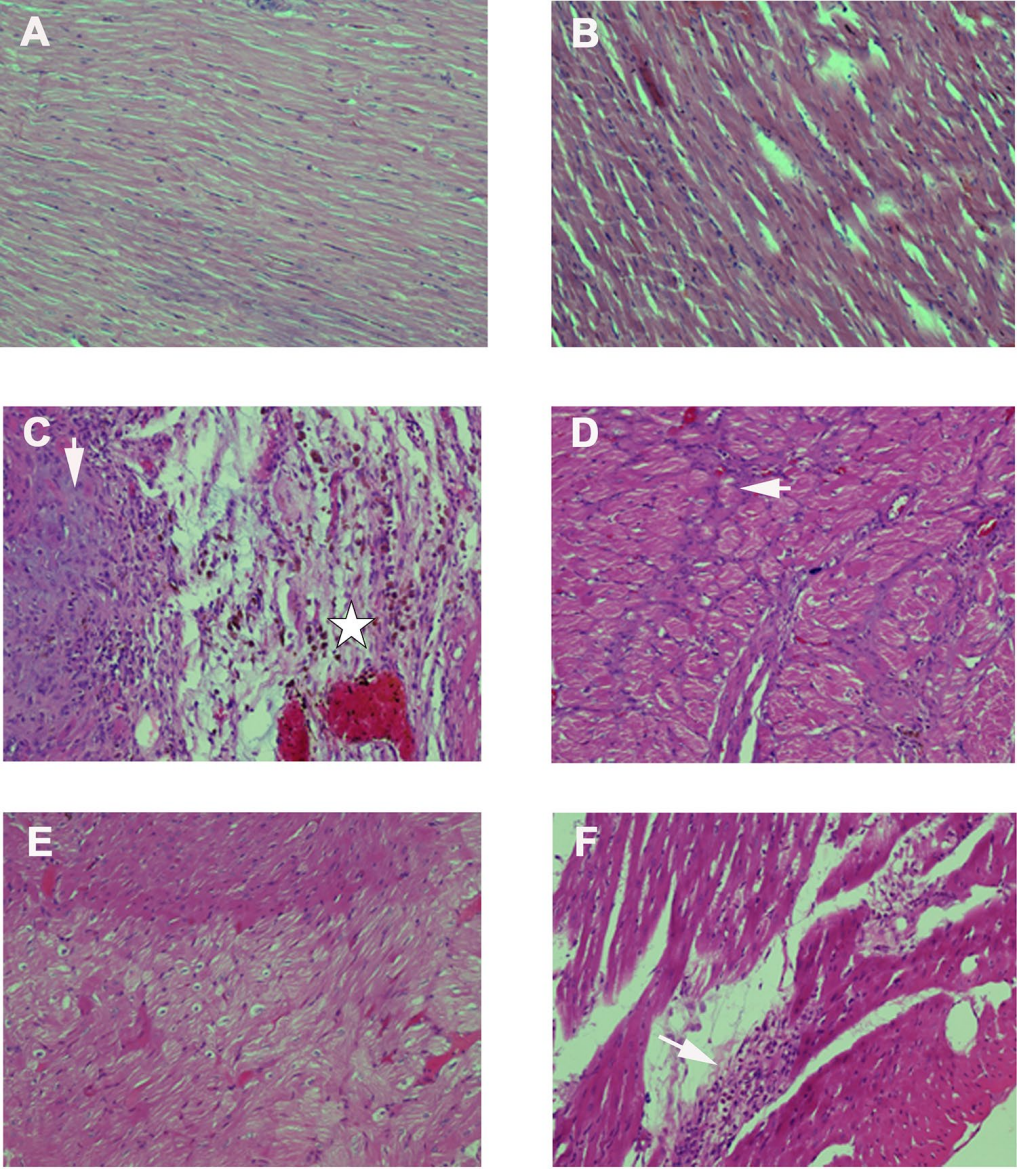
Histological analysis of myocardial injury on H&E stain. Histomicrograph of transverse sections of the heart [× 200] taken through the ventricles, just below the atrioventricular valves of Control (**A**), Mb (**B**), l-NAME (**C**), l-NAME + Mb (**D**), DOCA (**E**) and DOCA + Mb (**F**) groups.

Microscopic changes to the muscle fibers of the heart were identified in three groups; the l-NAME groups with and without exposure to Mb and the DOCA group that was exposed to the Mb (Fig. 6, Table 2). There was no significant myocardial hypertrophy, which was expected in long standing hypertension, instead there were areas of myocardial infarction that were most pronounced in the l-NAME groups. In the l-NAME groups the myocardial damage was multifocal and had a maximum dimension of 8.4 mm in the rats not subjected to the extract. Accompanying the myocardial injury was an infiltrate of chronic inflammatory cells, in particular lymphocytes and macrophages, which was quite severe in the l-NAME group and appeared to wane in the Mb treated group. Chronicity of injury in the l-NAME group was further evidenced by cardiac myocyte atrophy coupled with hydropic cytoplasmic change.

**Table 2:** Microscopic evaluation of *bijugatus* extract (Mb), l-NAME and DOCA-salt on cardiac tissue.

	Myocardial alterations					
	Inflammatory cell infiltrate	Acute changes		Chronic changes		
		Haemorrhage/edema	Pyknosis/karyolsis/eosinophilic change	Hyper-trophy	Atrophy/hydropic change	Fibrosis
Control	−	−	−	−	−	−
Mb	−	−	−	−	−	−
l-NAME	+++ (lymphocytes and macrophages)	+	+	−	+	Sub-endocardial region (× 2 foci—8.4 mm max dimension)
l-NAME + Mb	+ (lymphocytes and macrophages)	−	−	−	+	Intramural region (× 3 foci—1.8 mm)
DOCA	−	−	−	−	−	−
DOCA + Mb	−	−	−	−	−	Sub-endocardial region (× 1 focus—1.0 mm max dimension)

**Figure 7:**
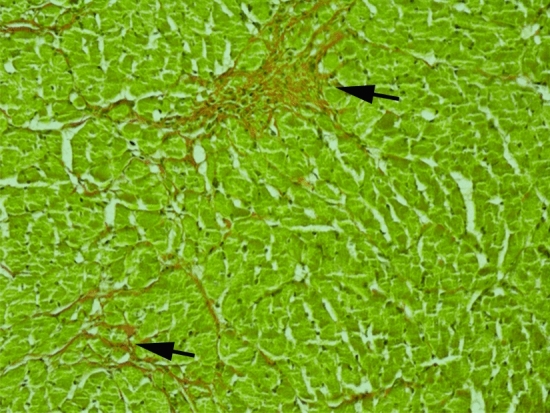
Histological analysis of myocardial injury on trichrome stain. Histomicrograph of transverse sections of the heart [× 200] taken through the ventricles, just below the atrioventricular valves of l-NAME + Mb treated group highlighting fibrosis and myocyte dropout (arrows).

As shown in Fig. 6, sections from the control, Mb and DOCA treated groups did not show abnormalities. The l-NAME group showed recent-on-remote myocardial infarction evidenced by mononuclear cell infiltration with oedema (star) and degeneration of the myocytes with fibrosis (arrow; C). The l-NAME + Mb treated group showed fibrosis, albeit, subtly with mild chronic inflammation (arrow; D; Fig. 7). The DOCA + Mb group demonstrated sub-endocardial fibrosis and myocyte degeneration indicative of infarction (arrow; F).

### Average body weight/g of the animals

Table 3 shows that there was no significant variance between average body weight and kidney weight. The administered extract had no effect on kidney or body weight. There was also, no significant variance between average body weight and heart weight. The administered extract had no effect on heart or body weight for the experimental l-NAME and DOCA-salt groups.

**Table 3:** Effect of *M. bijugatus* (Mb; 100 mg/kg) on body weight, heart weight ratio and kidney weight ratio in normotensive rats and hypertensive-induced models with l-NAME or DOCA-salt.

	Normotensive		Hypertensive models			
	Control	Mb	l-NAME	l-NAME + Mb	DOCA	DOCA + Mb
Body weight change (g)	18 ± 14	5.0 ± 5	50.0 ± 29	36 ± 25	40 ± 17	5 ± 0
Heart weight ratio (mg)	3.20 ± 0.23	2.70 ± 0.47	3.45 ± 0.32	3.36 ± 0.40	3.26 ± 0.25	3.46 ± 0.08
Kidney weight ratio (mg)	3.52 ± 0.17	3.72 ± 0.44	3.56 ± 0.30	3.22 ± 0.19	3.75 ± 0.15	4.01 ± 0.14

Values are mean ± standard error of the mean (SEM) of 4 experiments.

## Discussion

This study reported for the first time the antihypertensive and hypotensive properties of the aqueous extract of *M. bijugatus* in experimental hypertensive animal models, using in vitro and in vivo techniques to ascertain the mechanisms of action. The l-NAME experimental hypertensive model showed significantly elevated mean arterial pressure, while the treatment with *M. bijugatus* significantly reduced MAP and cardiac output. In normotensive animals, *M. bijugatus* extract caused significant reduction in MAP, which could be mediated by Peripheral Resistance (PR) and vasodilation, but not cardiac output. This result of the blood pressure indices is in keeping with our earlier reports on the effects of this extract^7^.

*Melicoccus bijugatus* reduced the MAP and DBP in DOCA-salt group, but not the SBP. Although there was no observed decrease in PR in DOCA + Mb group^14^, a significant reduction in cardiac output was observed. DOCA-salt rats provides an animal model of oxidative stress, inflammatory stress and hypertension in the cardiovascular system^15^. Which is due to DOCA stimulation of the Renin–Angiotensin–Aldosterone System (RAAS), and the sympathetic nervous system^16^. This stimulation increases DOCA-induced reabsorption of NaCl and water, which occurs from the stimulation of the brain RAAS, vasopressin release and vasoconstriction^17^. HR was significantly decreased in DOCA-salt group compared to control, which was probably due to an imbalance in sympathovagal versus direct effect of the mineralocorticoid on the sinus node^16^. The administration of *M. bijugatus* recovered the HR in DOCA-salt group, which is probably through a revision of the imbalance in sympathovagal effect, an observation similar to mechanisms reported for the extracts of *Mentha × villosa*^18^.

In the normotensive group, the decrease of HR, mediated by *M. bijugatus* also contributed to a decrease of the MAP. The bradycardia effect of *M. bijugatus* on the HR may not be due an imbalance of sympathovagal activities, as *M. bijugatus* did not alter HRV, suggesting the effect of extract on HR maybe on the automatic sinus node. This was also confirmed with the ECG analysis, were the extract was shown to have no significant effects compared with the control group.

We propose that the effects of *M. bijugatus* on hypertensive rats could be mediated by other cardiovascular mechanisms without a reduction in HR. The results imply an intrinsic myocardial mechanism, such as may cause a significant decrease of stroke volume (pressure pulse), leading to the decrease in cardiac output. Our current findings are consistent with our reported results with the extracts of *Xenophylum poposum* (Phil) V.A Funk in angiotensin II hypertensive mouse model^19^. Also consistent with this phenomenon observed in our study was the reported implications of neural and hormonal systems that may play a role in the regulation of blood pressure^20^.

In the l-NAME group, *M. bijugatus* significantly decreased the MAP by reducing the stroke volume (pulse pressure) and then cardiac output, but not the PR. The cardiac output reduction in l-NAME + Mb group was higher than that observed for DOCA + Mb, l-NAME and DOCA groups. Therefore, it is likely that PR increased in l-NAME + Mb group to counteract (autoregulation) the highly significant reduction of the cardiac output and avoid a drastic decrease of the MAP^21^. Cardiac output reduction after treatment with Mb showed a significantly lower PP compared to l-NAME group. The reduction in the pulse pressure is a reported mechanism of hypotensive ability as we have previously reported for *Allium sativum*^22^. It is possible that NO synthesis inhibition in vascular endothelium of the l-NAME-induced hypertension model increased the PP through a reduction in artery compliance^23^, while the treatment with Mb caused the opposite effect, decreasing the CO. In addition, NO inhibition in the l-NAME induced hypertension model causes an increase in the blood pressure via endothelial damage, NO reduction, oxidative stress and RAAS (involving an increased renin concentration and Ang-II)^24^. Since Ang II induces inflammation and oxidative stress in l-NAME hypertensive model^25^ and *M. bijugatus* did not present good antioxidant activity (data not shown), it is possible that some bioactive molecules of the extract could induce an increase of the PR in l-NAME group.

A limitation of this study is that large arteries should have been isolated from the hypertensive models to assess the in vitro effects of Mb on vascular function and direct Mb-induced vasorelaxation in hypertensive arteries. This would have given a clearer picture to the effects of the extract on vascular reactivity in our hypertensive animal models.

In normotensive animals, *M. bijugatus* caused a dose-dependent relaxation of aortic rings pre-contracted with PE. Relaxation of the aortic rings with intact endothelium was significantly greater when compared with aortic rings without endothelium, indicating that the vascular relaxation activity involved endothelium dependent mechanisms^26,27^, as well as NO and guanylyl cyclase activity, but not prostaglandin dependent activity. The aqueous extract of *M. bijugatus* did not show any vascular response per se in unstimulated vasculature. The vasodilation induced properties of the extract supports the reduction of the PR and MAP in normotensive animals.

In normotensive animals, our results showed that *M. bijugatus* reduced the vascular contractile response to PE in control group, which suggested that the extract could decrease the cytosolic Ca^2+^ on vascular smooth muscle cells in response to PE^19^. Therefore, the vasodilator effect of *M. bijugatus* could lead to decreased PR in normotensive rat, which could explain in part, the decrease of the MAP.

Several studies have demonstrated that calcium channel blockers prevent the increase in blood pressure and impaired vasodilation induced by l-NAME^28^. A similar effect was shown in the inhibition of Angiotensin-Converting Enzyme (ACE) in l-NAME hypertensive model^29^, but not in DOCA-salt hypertensive model^30^. The antihypertensive effect of medicinal plants are often through vasodilation, such as observed in the treatment of l-NAME induced hypertension with *Moringa oleifera* Lam^31^ or DOCA-salt induced hypertension with *Hancornia speciosa* Gomes^32^.

In this present study, we did not observe significant myocardial hypertrophy, which was expected in long standing hypertension. Instead there were areas of myocardial infarction, which was mostly pronounced in the l-NAME groups. This myocardial damage was multifocal. Accompanying the myocardial injury was an infiltrate of chronic inflammatory cells, in particular lymphocytes and macrophages, which was quite extensive in the l-NAME only group and appeared to wane when exposed to Mb extract. This was also an observation with the Creatine kinase muscle-brain (CKMB) cardiac biomarkers (Supplementary Information), which was significantly increased in the l-NAME group, but decreased with Mb treatment. Chronicity of injury in the l-NAME group was further evidenced by cardiac myocyte atrophy coupled with hydropic cytoplasmic change. Longitudinal and/or transverse sections of the large caliber abdominal blood vessels, i.e. aorta and caudal vena cava revealed no changes in the intima, media or externa layer. The adventitia was composed primarily of brown fat. No inflammation was appreciated (data not shown). There were also no significant differences in the biochemical assays of other cardiac biomarkers like; High-sensitivity C-reactive protein (HS-CRP + CRP), Creatine kinase muscle-brain (CKMB), concentration of cardiac troponin I (cTnL), myoglobin (Myo) (Supplementary Information) for the experimental groups.

In conclusion, *M. bijugatus* significantly decreases blood pressure in hypertensive in vivo model, which may be mediated by reductions in cardiac output. In normotensive animals, extract causes significant reduction in MAP mediated by PR and vasodilation, but not cardiac output. The extract possesses the in vitro ability to induce endothelium dependent vasodilation, which is dependent on guanylyl cyclase but not prostaglandins. *M. bijugatus* extracts significantly decreased l-NAME and DOCA-salt induced pathologies, hypertensive parameters as well as myocardial and hepatic injury. A significant reduction of MAP after *M. bijugatus* treatment was greater in the hypertensive models than normotensive rats, suggesting that *M. bijugatus* treatment was more protective in l-NAME-induced hypertensive, a form of cardio-protection^33^. Further work is envisaged to further delineate the mechanisms behind Mb antihypertensive actions.

## Materials and methods

### Drugs

The drugs used were l-phenylephrine hydrochloride (PE), Acetylcholine chloride (ACh), 1H-(1,2,4) oxadiazolo[4,3-a]quinoxalin-1-one (ODQ), *N*^G^-nitro-l-arginine methyl ester (l-NAME), and Deoxycorticosterone acetate (DOCA) which were bought from Sigma-Aldrich (St Luis, MO, USA). Except for indomethacin, the drugs were dissolved in distilled and deionized water (deionized water Millipore) and kept at 4 °C. The stock solution of indomethacin was dissolved in dimethyl sulfoxide (DMSO) (Merck, Germany).

### Plant material extraction and analysis

Guinep fruits were identified, collected and used for the study. The skin was removed to extract the jelly part. The jelly was further macerated to yield a solution. The solution was filtered, extracted and concentrated using a Freeze dry methodology and machine. The concentrated extract was stored in a capped container and refrigerated at − 4 °C until ready for use.

### Experimental animals

The study used male Sprague Dawley rats (8–10 weeks old) weighing between 170 and 230 g and was conducted in accordance with the Animal Scientific Procedures Act of 1986, and with the approval of the University of the West Indies/University Hospital of The West Indies/Faculty of Medical Science ethics committee (AN 06,15/16). The animals were housed in plastic cages at a room temperature of 22–25 °C and humidity of 45–51% and had access to tap water and food ad libitum. They were randomized and assigned into groups. The first group served as a control group and so did not receive any drug. Rats in the second group were administered 100 mg/kg of *M. bijugatus* extract daily for the 6 weeks via oral gavage. Rats in groups 3 and 4 were treated with l-NAME (45 mg/kg body weight) solution via oral gavage after which the group 3 rats were administered *M. bijugatus* extract daily for the six weeks and had water ad libitum. Finally, groups 5 and 6 underwent surgery where Deoxycorticosterone Acetate (DOCA) 21-day pellets were inserted intraperitoneally. After 21 days, group 5 rats were fed only chow and 0.9% sodium chloride solution ad libitum to complete the 6-week period while group 6 received the extract and 0.9% sodium chloride ad libitum for the 6-weeks period. The rats were weighed at least 3 times a week to record any fluctuation in weight.

### Blood pressure recordings

Blood pressure measurements were carried out on rats in our laboratory as earlier described^34^.

Systolic blood pressure (SBP), Diastolic blood pressure (DBP) and Heart rates (HR) were measured at the end of the experimental period by the tail cuff method (CODA) after a warming period in un-anesthetized rats (following a period of conditioning/acclimatization to blood pressure measurements). Pulse pressure (PP) was calculated using the SBP and the DBP as follows: PP = (SBP–DBP). Mean Arterial Pressure (MAP) was calculated using the formula: MAP = P_diastole_ + 1/3 (P_systole_ − P_diastole_).

Several studies have used the tail-cuff method and report a high correlation with the direct intra-arterial measurement of blood pressure in small animals^35,36^. In addition, the tail-cuff method allows blood pressure measurement in conscious animals, without compromise of cardiovascular regulation noted with use of anesthesia and the associated mortality of the surgery^37^. However, the tail-cuff method is not convenient in evaluating subtle fluctuations in blood pressure or HR variations in response to stimuli^38^.

### Heart rate variability (HRV) of the electrocardiogram (ECG)

Sympathovagal balance or the heart rate variability (HRV) of the ECG was determined according to the procedure reported by Cifuentes et al.^39^. The frequency bands: total power (P: 0–3 Hz), power low-frequency (LF: 0.20–0.75 Hz), and high-frequency (HF: 0.75–3.0 Hz).

### Isolated organ bath experiments

Vascular reactivity was evaluated according to Cifuentes, Paredes et al.^19,40^. Following the sacrifice of the animals by cervical dislocation, the aorta was separated and transferred to a Krebs–Ringer bicarbonate buffer (KRB) solution at 4 °C, (mM): 4.2 KCl, 1.19 KH_2_PO_4_, 120 NaCl, 25 NaHCO_3_, 1.2 MgSO_4_, 1.3 CaCl_2_, and 5 D-glucose (pH7.4). 3–4 mm rings were prepared, and cleaned of connective tissue, taking special care to avoid endothelial damage. After 30 min period of equilibration, the aortic rings were stabilized with KCl (60 mM) near-maximum contractions for 10 min. We maintained a passive tension of 1.0 g on the aorta, as determined to be the optimal resting tension for obtaining maximum active tension in our laboratory. For dose–response curves, cumulative concentrions of PE (10^–9^ to 10^–5^ M) was used, and for relaxation experiments, the aortic rings were pre-contracted with PE 10^–6^ M. In addition, the aortic tissue was pre-incubated for 20 min with l-NAME 10^–4^ M, ODQ 10^–6^ M or indomethacin 10^–5^ M.

### Histormorphological analysis

The tissues of interest were harvested and submerged in 10% neutral buffered formalin within 5 min so as to decrease the ischaemia time and to allow for adequate fixation. Post 72 h of fixation the tissues were processed, i.e. dehydrated and impregnated with paraffin wax. The wax blocks were then serially sliced at a thickness of 4 µm and placed on positively charged glass slides. The tissues were stained with haematoxylin and eosin (H&E) stain.

The microscopic analysis of the tissues from the Sprague–Dawley rats was done utilizing an electronic Nikon Eclipse Ci research microscope (Nikon Instruments Inc., Americas). The microscope is equipped with a mechanical stage, slide holding receptacle and graduated locator knobs. The measurements were taken via scrolling within both X and Y-axes with the translator knobs.

The sections from the heart were taken in the horizontal plane just beneath the atrioventricular valves. Eighteen sections were taken in total, i.e. three from each of the study groups. The cardiac muscle was evaluated for degenerative features, namely, inflammatory cell infiltration, haemorrhage, oedema, fibrosis and evidence of cardiac muscle (myocyte) death. Quantification analysis was done via measuring the maximum dimension of the degenerate areas and tabulating the foci present in the microscopic fields.

### Statistics

The results obtained from these experiments were expressed as mean ± standard error of mean. Statistical analysis of the data was performed using analysis of variance (ANOVA) where applicable followed by post-hoc Bonferroni test where P values < 0.05 were significant. In addition, the determination of the sensitivity (pD_2_) was performed using nonlinear regression (sigmoidal) via Graph Pad Prism software, version 5.0. (GraphPad Software, Inc., La Jolla, CA, USA). Statistical significance is set at *P* < 0.05.

## Supplementary information

Supplementary Information.

## Data Availability

The datasets generated during and/or analyzed during the current study are available from the corresponding author on reasonable request.

## References

[1] Liwa AC (2017). Pharmacognosy.

[2] Bystrom LM (2012). The potential health effects of *Melicoccus bijugatus* Jacq. fruits: Phytochemical, chemotaxonomic and ethnobotanical investigations. Fitoterapia.

[3] Juraschek SP, Guallar E, Appel LJ, Miller ER (2012). Effects of vitamin C supplementation on blood pressure: A meta-analysis of randomized controlled trials. Am. J. Clin. Nutr..

[4] Palacios J (2018). Ascorbate attenuates oxidative stress and increased blood pressure induced by 2-(4-hydroxyphenyl) amino-1,4-naphthoquinone in rats. Oxid. Med. Cell. Longev..

[5] Halberstein RA, Saunders AB (1978). Traditional medical practices and medicinal plant usage on a Bahamian island. Cult. Med. Psychiatry.

[6] Facey PC, Pascoe KO, Porter RB, Jones AD (1999). Investigation of plants used in Jamaican folk medicine for anti-bacterial activity. J. Pharm. Pharmacol..

[7] Nwokocha CR (2019). Modulatory effect of guinep (*Melicoccus bijugatus* Jacq) fruit pulp extract on isoproterenol-induced myocardial damage in rats. Identification of major metabolites using high resolution UHPLC Q-Orbitrap mass spectrometry. Molecules.

[8] Rodrigues-Lima F, Dairou J, Laurieri N, Busi F, Dupret JM (2011). Pharmacogenomics, biochemistry, toxicology, microbiology and cancer research in one go. Pharmacogenomics.

[9] Sim E, Abuhammad A, Ryan A (2014). Arylamine *N*-acetyltransferases: From drug metabolism and pharmacogenetics to drug discovery. Br. J. Pharmacol..

[10] Marczylo T, Ioannides C (1998). The substrate specificity of the rat hepatic cytosolic arylamine oxidase catalyzing the bioactivation of aromatic amines. Cancer Lett..

[11] Ames BN (1974). A combined bacterial and liver test system for detection and classification of carcinogens as mutagens. Genetics.

[12] Bighamian R, Hahn JO (2014). Relationship between stroke volume and pulse pressure during blood volume perturbation: A mathematical analysis. Biomed. Res. Int..

[13] Cifuentes F (2009). Chronic exposure to arsenic in tap water reduces acetylcholine-induced relaxation in the aorta and increases oxidative stress in female rats. Int. J. Toxicol..

[14] Schenk J, McNeill JH (1992). The pathogenesis of DOCA-salt hypertension. J. Pharmacol. Toxicol. Methods.

[15] Iyer A, Chan V, Brown L (2010). The DOCA-salt hypertensive rat as a model of cardiovascular oxidative and inflammatory stress. Curr. Cardiol. Rev..

[16] Guimaraes PS (2012). Chronic infusion of angiotensin-(1–7) into the lateral ventricle of the brain attenuates hypertension in DOCA-salt rats. Am. J. Physiol. Heart Circ. Physiol..

[17] Basting T, Lazartigues E (2017). DOCA-Salt hypertension: An update. Curr. Hypertens. Rep..

[18] Lahlou S, Carneiro-Leão RF, Leal-Cardoso JH (2002). Cardiovascular effects of the essential oil of Mentha × villosa in DOCA-salt-hypertensive rats. Phytomedicine.

[19] Cifuentes F (2018). Vasodilator and hypotensive effects of pure compounds and hydroalcoholic extract of *Xenophyllum poposum* (Phil) V.A Funk (Compositae) on rats. Phytomedicine.

[20] Collister JP, Hornfeldt BJ, Osborn JW (1996). Hypotensive response to losartan in normal rats. Role of Ang II and the area postrema. Hypertension.

[21] Pickering TG, Laragh JH (1980). Autoregulation as a factor in peripheral resistance and flow: Clinical implications for analysis of high blood pressure. Am. J. Med..

[22] Nwokocha CR, Ozolua RI, Owu DU, Nwokocha MI, Ugwu AC (2011). Antihypertensive properties of *Allium sativum* (garlic) on normotensive and two kidney one clip hypertensive rats. Niger. J. Physiol. Sci..

[23] Ruiz A, López RM, Pérez T, Castillo C, Castillo EF (2008). The effects of NG-nitro-l-arginine methyl ester on systolic pressure, diastolic pressure and pulse pressure according to the initial level of blood pressure. Fundam. Clin. Pharmacol..

[24] Ishiguro K, Sasamura H, Sakamaki Y, Itoh H, Saruta T (2007). Developmental activity of the renin-angiotensin system during the "critical period" modulates later l-NAME-induced hypertension and renal injury. Hypertens. Res..

[25] Maneesai P (2016). Synergistic antihypertensive effect of *Carthamus tinctorius* L. extract and captopril in L-NAME-induced hypertensive rats via restoration of eNOS and AT_1_R expression. Nutrients.

[26] Nwokocha CR, Nwokocha MI, Owu DU, Ajayi IO, Ebeigbe AB (2012). Experimental malaria: The in vitro and in vivo blood pressure paradox. Cardiovasc. J. Afr..

[27] Nwokocha CR (2012). Possible mechanisms of action of the aqueous extract of *Artocarpus altilis* (breadfruit) leaves in producing hypotension in normotensive Sprague-Dawley rats. Pharm. Biol..

[28] Küng CF, Moreau P, Takase H, Lüscher TF (1995). L-NAME hypertension alters endothelial and smooth muscle function in rat aorta. Prevention by trandolapril and verapamil. Hypertension.

[29] Zicha J, Dobesová Z, Kunes J (2006). Antihypertensive mechanisms of chronic captopril or *N*-acetylcysteine treatment in L-NAME hypertensive rats. Hypertens. Res..

[30] Peng H, Carretero OA, Alfie ME, Masura JA, Rhaleb NE (2001). Effects of angiotensin-converting enzyme inhibitor and angiotensin type 1 receptor antagonist in deoxycorticosterone acetate-salt hypertensive mice lacking Ren-2 gene. Hypertension.

[31] Aekthammarat D, Pannangpetch P, Tangsucharit P (2019). Moringa oleifera leaf extract lowers high blood pressure by alleviating vascular dysfunction and decreasing oxidative stress in L-NAME hypertensive rats. Phytomedicine.

[32] Silva GC, Braga FC, Lemos VS, Cortes SF (2016). Potent antihypertensive effect of *Hancornia speciosa* leaves extract. Phytomedicine.

[33] Nwokocha CR (2020). Protective effects of apocynin against cadmium toxicity and serum parameters; evidence of a cardio-protective influence. Inorg. Chim. Acta.

[34] Nwokocha C (2017). Aqueous extract from leaf of *Artocarpus altilis* provides cardio-protection from isoproterenol induced myocardial damage in rats: Negative chronotropic and inotropic effects. J. Ethnopharmacol..

[35] Krege JH, Hodgin JB, Hagaman JR, Smithies O (1995). A noninvasive computerized tail-cuff system for measuring blood pressure in mice. Hypertension.

[36] Daugherty A, Rateri D, Hong L, Balakrishnan A (2009). Measuring blood pressure in mice using volume pressure recording, a tail-cuff method. J. Vis. Exp..

[37] Wang Y, Thatcher SE, Cassis LA (2017). Measuring blood pressure using a noninvasive tail cuff method in mice. Methods Mol. Biol..

[38] Drüeke TB, Devuyst O (2019). Blood pressure measurement in mice: Tail-cuff or telemetry?. Kidney Int..

[39] Cifuentes F (2016). Hypotensive and antihypertensive effects of a hydroalcoholic extract from *Senecio nutans* Sch. Bip. (Compositae) in mice: Chronotropic and negative inotropic effect, a nifedipine-like action. J. Ethnopharmacol..

[40] Paredes A (2016). Hydroalcoholic extract and pure compounds from *Senecio nutans* Sch. Bip (Compositae) induce vasodilation in rat aorta through endothelium-dependent and independent mechanisms. J. Ethnopharmacol..

