# Supplementary material for: Hypotensive and antihypertensive effects of an aqueous extract from Guinep fruit (Melicoccus bijugatus Jacq) in rats
Source: Sci Rep. 2020 Oct 29;10:18623. doi: 10.1038/s41598-020-75607-3 (PMC7596469; doi:10.1038/s41598-020-75607-3)
Supplement: Supplementary file 1 — Supplementary Information. [file 41598_2020_75607_MOESM1_ESM.docx]

## Supplementary material

**Hypotensive and antihypertensive effects of an aqueous extract from Guinep fruit (*Melicoccus bijugatus* Jacq) in rats.**

Chukwuemeka R. Nwokocha^1*^, Alexia Gordon^1^, Javier Palacios^2**^, Adrian Paredes^3^, Fredi Cifuentes^4^, Sheena Francis^5^, JeAnn Watson^5^, Rupika Delgoda^5^, Magdalene Nwokocha^6^, Ruby Alexander-Lindo^1^, Rory Thompson^6^, Donna Minott Kates^7^, Momoh A. Yakubu^8^

^1^Department of Basic Medical Sciences, Faculty of Medical Sciences, The University of the West Indies, Mona Campus, Kingston 7, Jamaica.

^2^Facultad de Ciencias de la Salud, Instituto de EtnoFarmacología (IDE), Universidad Arturo Prat, 1110939 Iquique, Chile.

^3^Laboratorio de Química Biológica Instituto Antofagasta, Universidad de Antofagasta, 1270300 Antofagasta, Chile.

^4^Laboratorio de Fisiología Experimental Instituto Antofagasta, Universidad de Antofagasta, 1270300 Antofagasta, Chile.

^5^Natural Products Institute, Faculty of Science and Technology, The University of the West Indies, Mona, Kingston 7, Jamaica.

^6^Department of Pathology, Faculty of Medical Sciences, University of the West Indies, Mona Campus, Kingston 7, Jamaica.

^7^Department of Chemistry, University of The West Indies, Mona, Kingston 7, Jamaica.

^8^Department of Environmental and Interdisciplinary Sciences, College of Science, Engineering and Technology, Texas Southern University, Houston, TX 77004, USA.

Correspondence: *[chukwuemeka.nwokocha@uwimona.edu.jm](mailto:chukwuemeka.nwokocha@uwimona.edu.jm) (C.R.N.); **[clpalaci@unap.cl](mailto:clpalaci@unap.cl) (J.P.); Tel.: +18765895445 (C.R.N.); +56-57-2526910 (J.P.)

- 1. *Histormorphological analysis of liver*

The liver was sectioned in the vertical plane irrespective of the anatomic segment. Twelve sections were taken in total, two from each of the study groups. The hepatic parenchyma was analyzed for inflammation, cholestasis, steatosis and fibrosis. The stage of fibrosis and grade of inflammation were specifically quantified using the Ishak system ^1^. The level of steatosis was ascribed a grade based on a schema utilizing Kleiner-Brunt scores ^2^. Cholestasis scoring is in the rudimentary stages and the proposed system highlighted by Dixon & Crawford ^3^ was utilized in this study. There are no universally accepted grading schemes for sinusoidal congestion and nuclear chromatin density, therefore, semi-objective parameters were utilized.

Sections from the Control, DOCA, L-NAME and Mb treated groups showed no significant abnormalities (**Figure 1S** and **Table 1S**). The DOCA+Mb treated group demonstrated sinusoidal congestion and vascular congestion (arrows; **C**) only. The L-NAME+Mb treated group showed confluent necrosis effacing the zonal architecture of the liver (stars; **E**).

| **Table 1S: Microscopic evaluation of *M. bijugatus* extract (Mb), L-NAME and DOCA-salt on liver tissue.** | | | | | | | | |
| --- | --- | --- | --- | --- | --- | --- | --- | --- |
|  | **HEPATIC ALTERATIONS** | | | | | | | |
|  | FIBROSIS | CHOLESTASIS | STEATOSIS | SINUSOIDAL CONGESTION | INFLAMMATION & NECROSIS | | | |
|  |  |  |  |  | CONFLUENT | INTERFACE | LOBULAR | PORTAL |
| CONTROL | - | - | - | - | - | - | - | - |
| Mb | - | - | - | - | - | - | - | - |
| L-NAME | - | - | - | - | - | - | - | - |
| L-NAME+Mb | - | - | - | + | +++ | - | - | + |
| DOCA | - | - | - | - | - | - | - | - |
| DOCA+Mb | - | - | - | + | - | - | - | - |

The most significant microscopic change in the liver was that of confluent necrosis, which was, present within the L-NAME+Mb treated group. The necroinflammation extended to involve all zones of the liver and was associated with a mild periportal inflammatory cell infiltrate away from the areas of necrosis. This group along with the DOCA+Mb group also demonstrated mild sinusoidal congestion which was indicative of poor cardiac function and early right-sided heart failure. There was no fibrosis, or changes to the density of nuclear chromatin seen. Fat deposition within hepatocytes (steatosis) was not appreciated neither was there any cholestasis which would have been indicative of bile duct obstruction and hepatocyte damage.

**
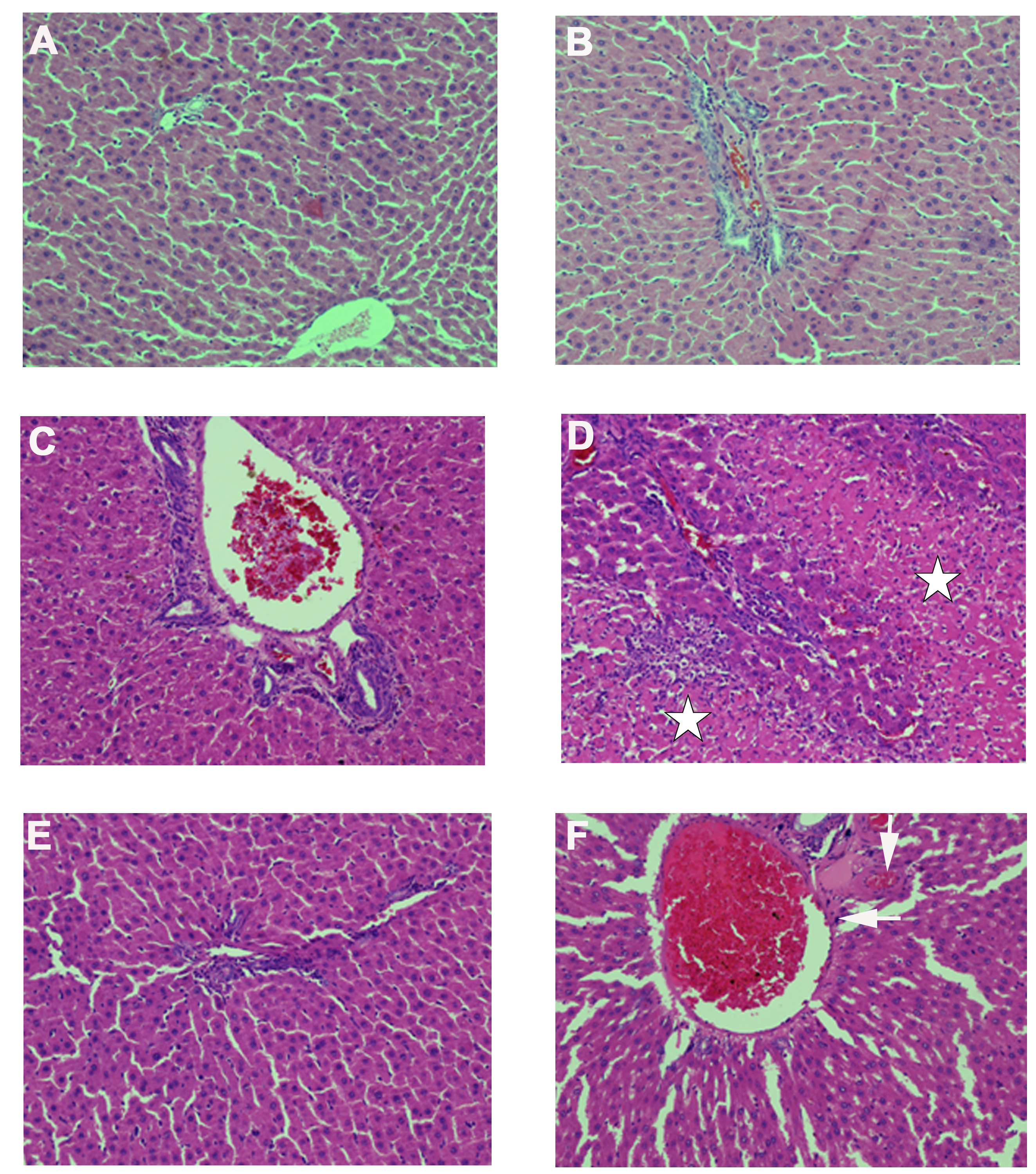
**

**Figure 1S.** Histological analysis of hepatic alterations on H&E. Histomicrograph of random sections of the liver [x200] of Control (**A**), Mb (**B**), L-NAME (**C**), L-NAME+Mb (**D**), DOCA (**E**), and DOCA+Mb (**F**) treated groups.

1. *Protein Quantification in rat liver*

S9 fractions were prepared from homogenised rat liver that was previously washed in PBS and homogenised, 25% w/v in chilled KPB (10 mM; pH 7.4), containing 0.15 M KCl and 0.1 mM PMFS protease inhibitors (Roche). The homogenates were centrifuged at 9000g at a temperature of 4°C for 20 min. The amounts of proteins in all S9 fractions were prepared from liver homogenates determined using a standard Bradford Assay (BioRad, CA, USA) in triplicates, as described ^4^.

1. *Determination of Arylamine N-Acetyltransferase Activity*

The activities of Arylamine N-Acetyltransferase (NAT) in crude liver homogenates were conducted using p-ansidine (pANS), an ideal rat NAT substrate, as described ^5^. Briefly, S9 liver fractions were diluted so that NAT activity was in linear range with 40 µL of 1mM pANS, in 20 mM Tris-HCl, 10 mM NaCl, 1 mM EDTA, pH 7.5. 1 mM Acetyl coenzyme A (40 µL) was added to start the reaction; the final volume of the reaction was 200 µL. The reaction was allowed to incubate for 30 minutes at 37 °C after which it was terminated by adding the precipitating agent trichloroacetic acid [100 µL; 30% (w/v)] previously chilled to 4 °C. The reaction mixture was subsequently centrifuged to remove precipitated protein and then observed by spectrophotometry by adding 200 µL of 5% (w/v) dimethylaminobenzaldehyde (DMAB). Unacetylated pANS was detected using a UV-spectrophotometer (µQuant universal microplate spectrophotometer, Bio-Tek Instruments, Winooski, VT, USA) at 450 nm.  Enzymatic NAT activity values are expressed as µmols of N-acetylated p-ANS/min/µg of liver protein. All reactions were conducted in triplicates, and in accordance to principles of identifications of bioactive samples and mechanisms of actions ^6^.

There appears to be neither significant enzyme inhibition nor induction in the presence of the *M. bijugatus* extract, indicative of the absence of toxicity arising from acetylation mediated activation of arylamine and hydrazine based endogenous and exogenous chemicals (**Figure 9**). Combination treatment of DOCA-salt with Mb did not change NAT activity when compared to DOCA-salt alone, but both DOCA-salt alone (0.164 ± 0.017 μg/min/μg protein; P <0.05) and in combination with Mb (0.147 ± 0.026 μg/min/μg protein) showed higher NAT activities than that in the control (0.073± 0.016 μg/min/μg protein). The lowest enzyme activity was detected in the group treated with L-NAME (0.066 ± 0.015 μg/min/μg protein), which was comparable to that of the control.

**
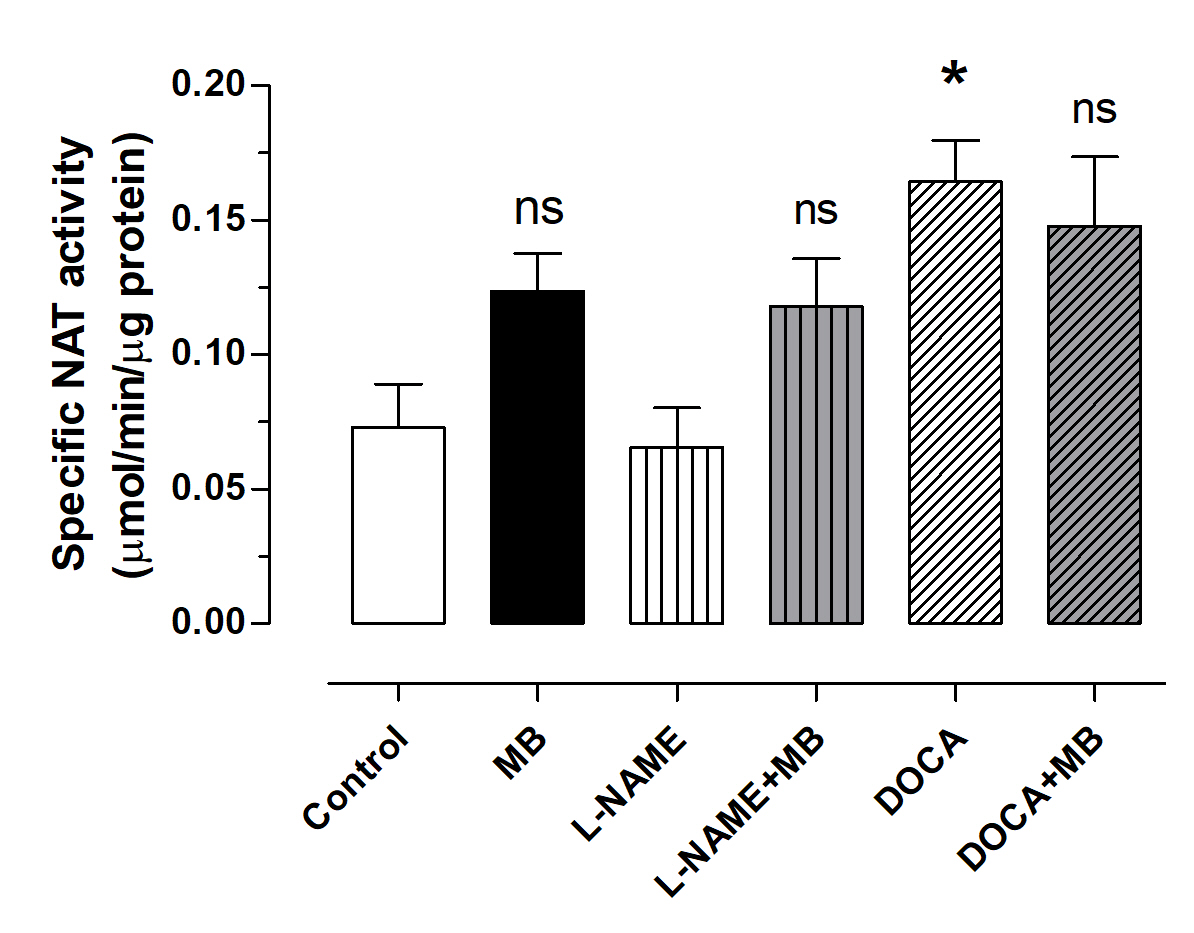
**

**Figure 2S.** Arylamine N-acetyltransferase activity on animal treatment. Enzymatic NAT activity values are expressed as µmol of *N*-acetylated *p*-ANS/min/µg of liver protein. Each data point represents the mean ± SEM. Not significant (ns) versus Control, L-NAME or DOCA, respectively. **P <* 0.05 vs. Control. *n* = 6

The NATs enzymes are involved in the [metabolism](https://en.wikipedia.org/wiki/Metabolism) of [xenobiotics](https://en.wikipedia.org/wiki/Xenobiotic) by catalytic transfer of an acetyl group from acetyl-CoA to the terminal nitrogen of the antihypertensive agent hydralazine and arylamine drugs as well as carcinogens ^7^. This phase II metabolic process by NAT can lead to detoxification and inactivation of drugs as well as bioactivation of drugs/chemicals into toxic metabolites, which may be carcinogenic ^7^. In the present study, we observed that treatments with Mb extract alone or DOCA-salt alone but not L-NAME increased NAT activity when compared to the control. The combination treatment of L-NAME and Mb did not have any effect on NAT when compared with Mb alone but was higher than that observed in L-NAME alone. Combination treatment of DOCA-salt with Mb did not change NAT activity when compared to DOCA-salt alone, but both DOCA-salt alone and in combination with Mb were still higher than that in the control. The implication of these findings may not necessarily mean that Mb may be toxic by induction of NAT activity, but it could be that the presence of Mb and/or DOCA in the biological system are triggers for the normal metabolism of these xenobiotics. Such may not necessarily lead to bioactivation of these xenobiotics to produce toxic reactions in the body-consistent with this idea is the fact that Mb has shown the ability to ameliorate the cardiovascular effects of the DOCA salt. Furthermore, treatment of normal cell line in culture with Mb extract did not show any adverse effects on cell viability as assessed by MTT assay (results not shown). We can therefore say that consumption of *M. bijugatus* does not appear to cause any deleterious impact at the cellular level over a short period of time but is effective in alleviating hypertension induced by L-NAME and DOCA-salt.

| 1. *HS-CRP + CRP/ CK-MB/ cTnI / Myo Immunofluorescence Quantitative Assay for Cardiac damage*   An immunofluorescence Assay was conducted using the Getein1100 Immunofluorescence Quantitative Analyzer (China) to determine the creatine kinase-muscle/brain, Myoglobin, Troponin I (CK-MB/cTnI/Myo) concentration *in vitro*. Elevated serum levels of Creatine Kinase and Troponin I are used as diagnostic markers of myocardial tissue damage. The test card, serum sample and reagent were brought to room temperature. The Getein 1100 was calibrated. A new test card was removed from the pouch immediately before use and labelled with the animal identification. The test card was then placed horizontally on a clean table. Using the sample transfer pipette, 100µl of sample (or 3-4 drops when using a disposable pipette) was transferred into the sample port on the test card. Ten minutes were allowed to elapse to give the reaction time to occur. Following this the test card was inserted into the Getein1100 and “ENT” button was pressed to obtain the results.  **Table 2S. Effect of *M. bijugatus*  (Mb; 100 mg/kg) on cardiac biomarkers in normotensive on rats and hypertensive-induced models with L-NAME or DOCA-salt.** | | | | | | |
| --- | --- | --- | --- | --- | --- | --- |
|  | **Normotensive** | | **Hypertensive models** | | | |
|  | **Control** | **Mb** | **L-NAME** | **L-NAME+Mb** | **DOCA** | **DOCA+Mb** |
| **HS-CRP + CRP (mg/L)** | 0.5 ± 0.0 | 0.5 ± 0.0 | 0.5 ± 0.0 | 0.5 ± 0.0 | 0.5 ± 0.0 | 0.5 ± 0.0 |
| **CKMB <2.50 ng/ml** | 2.5 ± 0.0 | 2.50 ± 0.0 | 43.5 ± 11.3*** | 13.1 ± 1.6## | 2.5 ± 0.1 | 8.3 ± 6.0 |
| **cTnL ≤0.01 ng/ml** | 0.07 ± 0.06 | 0.04 ± 0.03 | 0.01± 0.01 | 0.01 ± 0.01 | 0.03 ± 0.02 | 0.01± 0.01 |
| **Myo ≤ 30.0 ng/ml** | 30 ± 0 | 30 ± 0 | 30 ±0 | 30 ± 0 | 30 ±0 | 30 ± 0 |
| Values are mean ± standard error of the mean (SEM) of 4 experiments. ****p* < 0.001 versus Control; ##*p* < 0.01 versus L-NAME. | | | | | | |

Creatine kinase is an intracellular enzyme present in greatest amounts in skeletal muscle, myocardium, and brain. Injury to the myocardium due to hypoxia causes the membranes of the cardiac myocytes to become leaky and release creatine kinase from the cellular cytosol into the systemic circulation. Following the onset of symptoms of myocardial infarction CK and CK–MB (creatine kinase- muscle/brain) will increase in concentration in the serum within 3 to 6 hours as these enzymes continuously leak from the myocyte cytosol. The peak levels occur between 16 and 30 hours after the first symptoms are felt.

Troponin I also known as cTnL is the contractile protein that is found exclusively in the heart. Injury to the myocardial cells cause the cells’ membranes to destabilize and become leaky and release Troponin I from the cellular cytosol into the systemic circulation. Troponin I levels stay elevated for a week after the onset of cardiac muscle damage before returning to basal levels in the serum. Results indicated that there was not a significant elevation in serum Troponin I, creatine kinase muscle/brain, myoglobin, high sensitivity C- reactive protein in the experimental groups. It may also be that these substances had become elevated, but their levels had subsided within the time preceding the test.

In conclusion, there were also no significant differences in the biochemical assays of High-sensitivity C-reactive protein (HS-CRP+CRP), Creatine kinase muscle-brain (CKMB), concentration of cardiac troponin I (cTnL), myoglobin (Myo) (**Table 2S**) in all the experimental groups.

**References**

1 Goodman, Z. D. Grading and staging systems for inflammation and fibrosis in chronic liver diseases. *Journal of Hepatology* **47**, 598-607, doi:10.1016/j.jhep.2007.07.006 (2007).

2 Kleiner, D. E. *et al.* Design and validation of a histological scoring system for nonalcoholic fatty liver disease. *Hepatology* **41**, 1313-1321, doi:10.1002/hep.20701 (2005).

3 Dixon, L. R. & Crawford, J. M. Early histologic changes in fibrosing cholestatic hepatitis C. *Liver Transplantation* **13**, 219-226, doi:10.1002/lt.21011 (2007).

4 Francis, S. *et al.* Insecticide resistance to permethrin and malathion and associated mechanisms in Aedes aegypti mosquitoes from St. Andrew Jamaica. *PloS one* **12**, e0179673, doi:10.1371/journal.pone.0179673 (2017).

5 Francis, S., Laurieri, N., Nwokocha, C. & Delgoda, R. Treatment of rats with apocynin has considerable inhibitory effects on arylamine N-acetyltransferase activity in the liver. *Scientific reports* **6**, 26906 (2016).

6 McNeil, M. J. *et al.* Chemical composition and biological activities of the essential oil from Cleome rutidosperma DC. *Fitoterapia* **129**, 191-197, doi:10.1016/j.fitote.2018.07.006 (2018).

7 Sim, E. *et al.* Arylamine N-acetyltransferases: a pharmacogenomic approach to drug metabolism and endogenous function. *Biochem Soc Trans* **31**, 615-619, doi:10.1042/bst0310615 (2003).
